# Direct power control and back-stepping-based direct power control for dual-stator brushless doubly-fed wind power generator

**DOI:** 10.1371/journal.pone.0329181

**Published:** 2025-08-07

**Authors:** Xiaoying Su, Liancheng Zhu, Fengge Zhang

**Affiliations:** 1 School of Electrical Engineering, Shenyang University of Technology, Shenyang, China; 2 School of Electronic and Information Engineering, University of Science and Technology Liaoning, Anshan, China; 3 School of Electrical Engineering, Liaoning University of Technology, Jinzhou, China; University of Botswana Faculty of Engineering and Technology, BOTSWANA

## Abstract

This paper proposes a novel-structured Lyapunov-based back-stepping direct power control (BS-DPC) for an emerging dual-stator brushless doubly-fed wind power generator (DSBDFWPG). The DSBDFWPG features two coaxial stators (inner/outer) and a specially designed cage-barrier rotor separated by a non-magnetic ring, maximizing the internal space utilization of large-scale wind turbines to enhance power and torque density. The inner/outer power/control windings couple with corresponding rotor sections, respectively. Unlike traditional look-up table DPC (LUT-DPC) or generic nonlinear methods, the proposed BS-DPC is rooted in Lyapunov stability theory, ensures global asymptotic stability, while synergizing with SVPWM technology to suppress power fluctuations, reduce current THD, and enhance reliability/robustness. Comprehensive simulations and experiments conducted on a 50 kW DSBDFWPG prototype (12/8 poles) validate BS-DPC’s superiority overwhelming LUT-DPC in achieving variable speed constant frequency operation, maximum power point tracking, reactive power/unit power factor control, and harmonic distortion mitigation.

## Introduction

Wind power generation has achieved substantial global growth including economically developed and underdeveloped countries in recent years [1, 2]. The world wind power generation is developing towards offshore wind power generation system (WPGS) due to its huge wind energy reserves, which requires higher unit capacity, power density, reliability and performance-price ratio of generators and their control systems. As a member of slip power recovery family, the emerging brushless doubly-fed generators (BDFGs) evolved from two cascaded induction motors, but different from the conventional doubly-fed induction generators (DFIGs). The BDFGs have eliminated the brushes and slip-rings with moving the rotor winding to the stator, which currently consist of two robust standard three-phase sinusoidal distributed stators with different pole numbers and applied frequencies, and a specially designed rotor having half of the total numbers of stator poles, normally equipped with a two-stage gearbox superior to DFIGs three-stage one [3–6]. Similar to the DFIGs, but without reliability problems of brushes and slip-rings and almost maintenance-free for long operation, BDFGs only require partially-rated converter approximately 1/4–1/3 of nominal BDFGs capacity for typically limited speed ratio of 3:1 or 2:1, which have the overwhelming advantage over fully-rated converter of permanent magnet synchronous generators (PMSGs) [7]. Moreover, thanks for the larger leakage inductance of BDFG with reluctance rotor, which can lower low-voltage-ride-through-fault current than the DFIGs and PMSGs, without auxiliary Crowbar circuits [8–10]. BDFG’s advantages position it as a strong competitor to widely-used DFIGs and PMSGs in variable speed constant frequency (VSCF) WPGS.

So far, many typical control methods and strategies developed from DFIGs have been applied to BDFGs, such as scalar control (SC), vector control (VC) or field-oriented control (FOC), direct torque control (DTC) and direct power control (DPC). SC is simple but cannot meet the higher performance requirements of VSCF WPGS [11]. Sensored [6,11–14] or sensorless [7,15–17] VC/FOC requires complex rotational coordinate transformation, flux (field) orientation, accuracy mathematical model and parameters, which make the controller under large calculation pressure. In [18], a complex vector model for the dual-stator brushless doubly-fed induction generator (BDFIG) featuring a staggered dual-cage rotor is established, and a dual-loop controller with inner current and outer power loops is constructed, and validated its effectiveness via experimental tests showing close agreement with simulation results. Sensored [19] or sensorless [11,20] DTC only requires simple stator coordinate transformation, uses directly measured power winding voltage and current to calculate the electromagnetic torque and flux, with hysteresis comparator to complete the electromagnetic torque and flux self-control. In [21], to mitigate severe torque ripple from power winding open-phase faults in BDFIG-DC systems, a hardware-free minimum-ripple strategy is proposed, regulating power winding’s 3rd/5th harmonic currents via Newton’s gradient descent, and experimentally validated on a 5-kVA platform. DPC is a novel control method developed from DTC and instantaneous reactive power theory [22–24]. Similar to DTC, neither has the disadvantage of VC described above. The DPC replaces electromagnetic torque and flux of DTC with power winding active and reactive powers particularly concerned in WPGS, by synthesizing the output of power hysteresis comparators and flux sector of control winding, then the voltage vector of the machine side converter (MSC) is obtained by look-up table, and the active and reactive powers can be controlled in time. Moreover, by setting the reactive power reference value to 0, the unit power factor control (UPFC) of power winding can also be realized. In addition, the reliability and robustness of DPC can be further enhanced by adopting sensorless active and reactive power control strategy [25,26].

Furthermore, in [27], an extended vector control method is proposed for brushless doubly-fed reluctance generator under unbalanced grid conditions, enabling independent control of positive/negative sequence currents in the converter-fed winding. Its effectiveness in mitigating oscillations and improving current, torque, and power quality are validated through hardware-in-loop testing, demonstrating significant potential for grid integration of this emerging technology. In [28], to address system uncertainties and produce smooth control signals without requiring prior knowledge of uncertainty bounds, a dynamic sliding mode controller with adaptive gains is proposed for BDFIG in WPGS. An extended state observer is integrated to reduce derivative data requirements, with finite-time stability proven and effectiveness demonstrated through computer simulations on a detailed BDFIG model. In [29], an integrated dual-layer control strategy is proposed, comprising robust sliding mode control with maximum power point tracking (MPPT) for a brushless dual-fed induction generator, and synergetic control for flywheel energy storage management. Controller parameters are optimized via the Grasshopper Optimization Algorithm, achieving significantly improved dynamics and reduced tracking errors, with effectiveness validated under variable wind conditions through MATLAB/Simulink simulations.

This paper consists of the following parts. A general overview of WPGS development, BDFGs and their control technologies is given in Section 1. The stator and rotor structure, operating principles of VSCF, MPPT and dynamic mathematical model of the novel dual-stator brushless doubly-fed wind power generator (DSBDFWPG), with a back-to-back cage-barrier rotor are constructed in Section 2. The mechanisms of look-up table DPC (LUT-DPC) for DSBDFWPG and its optimized back-stepping-based DPC (BS-DPC) strategy are proposed and elaborated in Section 3. The comprehensive simulation and experimental results validate BS-DPC superior to LUT-DPC are implemented and analyzed in Section 4. Finally, Section 5 draws conclusion.

## Fundamentals of DSBDFWPG in VSCF-WPGS

### Stator and rotor structure of DSBDFWPG

**Fig 1:**
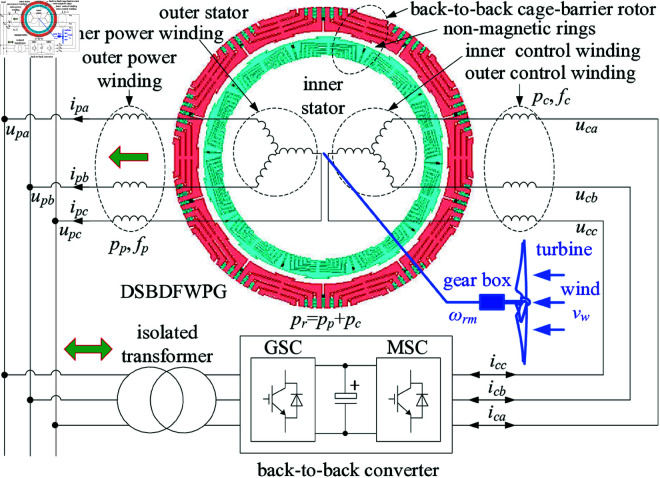
Topology of DSBDFWPG in VSCF-WPGS.

**Fig 2:**
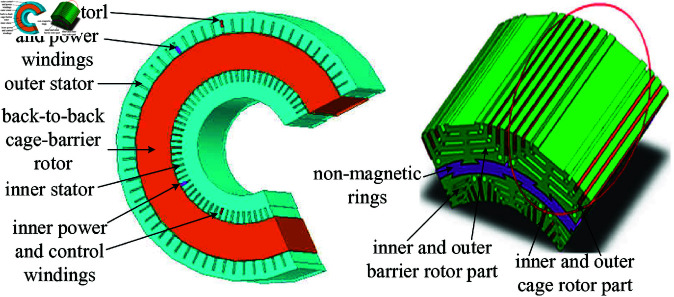
Structure schematic diagram of DSBDFWPG. (a) Stator. (b) Rotor.

The DSBDFWPG for VSCF WPGS is shown as Fig 1, where the two stators (in Fig 2(a)) consist of inner power (pole pairs and frequency as *p*_*p*_, *f*_*p*_) and control (*p*_*c*_, *f*_*c*_, and *p*_*p*_ > *p*_*c*_) windings and outer power (*p*_*p*_, *f*_*p*_) and control (*p*_*c*_, *f*_*c*_) ones, which coupled with a specially designed back-to-back inner and outer cage-barrier rotor (*p*_*r*_ = *p*_*p*_ + *p*_*c*_ for lower speed operation in VSCF WPGS) separated by the non-magnetic ring (in Fig 2(b)), respectively. To ensure electromagnetic consistency of the inner and outer same-phase stator winding, the inner and outer power/control windings are connected in series, respectively. The power winding is directly grid-connected, the control is fed with a back-to-back four-quadrant converter, allowing for bidirectional power flow under super-synchronous or sub-synchronous operation mode similar to the DFIGs, i.e., MSC and grid side converter (GSC).

The stator and rotor structure schematic diagram of DSBDFWPG as shown in Fig 2. Fig 2(a) includes inner power/control winding and outer power/control one, which coupled with the inner and outer parts of the back-to-back cage-barrier rotor, respectively. Fig 2(b) shows that an appropriate number of concentric paralleled short-circuit copper bars are nested to the inner and outer radially laminated magnetic-barrier reluctance rotor, which can comprehensively utilize the advantages of reluctance and cage rotor, then the overall performance of DSBDFWPG can be effectively promoted. It should be noted that the magnetic-barrier reluctance rotor plays a leading role, and the concentric cage rotor only as the assisted one. This particular structure described above can improve the magnetic field coupling ability between the inner/outer power and control windings, thus enhance the power density and efficiency characteristics of the DSBDFWPG [30–33].

### DSBDFWPG suitability for VSCF-WPGS

As shown in Fig 1, under the “sum modulation” (*p*_*r*_ = *p*_*p*_ + *p*_*c*_), the relationship among speed *n*_*r*_ (r/min), pole pairs (*p*_*p*_, *p*_*c*_) and frequencies (*f*_*p*_, *f*_*c*_) of the power and control windings are shown as (1), (2) [7]:

$$f_{p}=\frac{n_{r}p_{r}}{60}\pm f_{c}=\frac{n_{r}(p_{p}+p_{c})}{60}\pm f_{c}$$ (1)

$$\omega_{rm}=\frac{\omega_{p}+\omega_{c}}{p_{r}}=\frac{\omega_{p}}{p_{r}}(1+\frac{\omega_{c}}{\omega_{p}})=\omega_{syn}(1+\frac{\omega_{c}}{\omega_{p}})$$ (2)

where the subscripts “*p*", “*c*" and “*r*" indicate stator and rotor, respectively; $\omega_{p,c}$ and $\omega_{rm}$ (rad/s) denote the power and control angular frequencies corresponding to *f*_*p*,*c*_ and rotor mechanical angular velocity [7], i.e., $\omega_{p,c}\;=\;2\pi f_{p,c}$, $\omega_{rm}\;=\;2\pi n_{r}/60;\omega_{syn}\;=\omega_{p}/p_{r}$ (also $n_{syn}\;=\;60f_{p}/p_{r}$) presents the synchronous velocity with $\omega_{c}=0$ (i.e., the control winding is supplied with a DC link for *f*_*c*_ = 0), which similar to the wound rotor synchronous generator with *p*_*r*_ pole numbers; If $n_{r}>n_{syn}$ ($\omega_{r}>\omega_{syn}$), the generator operates in super-synchronous mode, otherwise, $n_{r}<n_{syn}$ ($\omega_{r}<\omega_{syn}$) for sub-synchronous mode. Obviously, it can be seen that when *n*_*r*_ changes with wind speed ($v_{w}$), it only needs to adjust the frequency and phase sequence of the control winding through MSC according to (1), then the frequency of power winding (*f*_*p*_) can be kept constant as line frequency 50 Hz of grid, i.e., the DSBDFWPG is very suitable for VSCF-WPGS [9,12,25,26].

### MPPT mechanisms of DSBDFWPG

On the basis of Betz’s law, the characteristics of wind turbine below the rated wind speed can be expressed as (3)–(7) [34]:

$$P_{m}=\frac{1}{2}C_{p}\rho \pi R^{2}v_{w}^{3}$$ (3)

$$C_{p}(\lambda ,\beta )=0.5176(\frac{116}{\lambda_{i}}-0.4\beta -5)e^{-\frac{21}{\lambda_{i}}}+0.0068\lambda$$ (4)

$$\lambda =\frac{\omega_{t}R}{v_{w}}$$ (5)

$$\frac{1}{\lambda_{i}}=\frac{1}{\lambda +0.08\beta }-\frac{0.035}{\beta^{3}+1}$$ (6)

$$n_{r}=Nn_{t}$$ (7)

where *P*_*m*_, *C*_*p*_, *ρ*, *R*, $v_{w}$, *λ*, *β*, *n*_*t*_, $\omega_{t}$ and *N* denote the mechanical power (kW) captured by wind turbine, wind power capture coefficient, air density (kg/$m^{3}$), radius of wind wheel (m), wind speed (m/s), tip speed ratio, pitch angel (°), rotor speed (r/min) and mechanical angular velocity (rad/s) of turbine and ratio of gear box, respectively.

**Fig 3:**
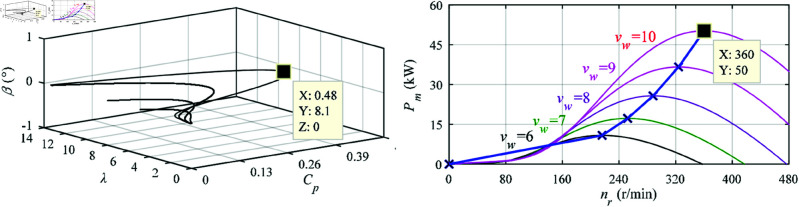
Characteristic curves of wind turbine. (a) $C_{p}-\lambda$. (b) $P_{m}-n_{r}$.

Setting the parameters of wind turbine as follows: start-up, rated and range of $v_{w}$ as 3.5 m/s, 10 m/s and 3.5–18 m/s, *R* = 7.5 m, *ρ* = 1.184 kg/$m^{3}$, *N* = 3.492. The characteristic curves of the wind turbine are shown in Fig 3. Noting that *β* should be minimum to capture the maximum wind energy as illustrated in Fig 3(a), i.e., *C*_*p*_ = 0.48, λ=8.1 and *β* = 0 for $v_{w}\leq 10$; meanwhile, the *n*_*r*_ of DSBDFWPG should be adjusted by *f*_*c*_ and phase sequence according to (1) for each value of different MPPT as illustrated in Fig 3(b), where *β* = 0, *C*_*p*_ = 0.48 and λ=8.1 for $v_{w}=10$, the rated power of wind turbine is 50 kW.

### Dynamic modelling of DSBDFWPG

**Fig 4:**
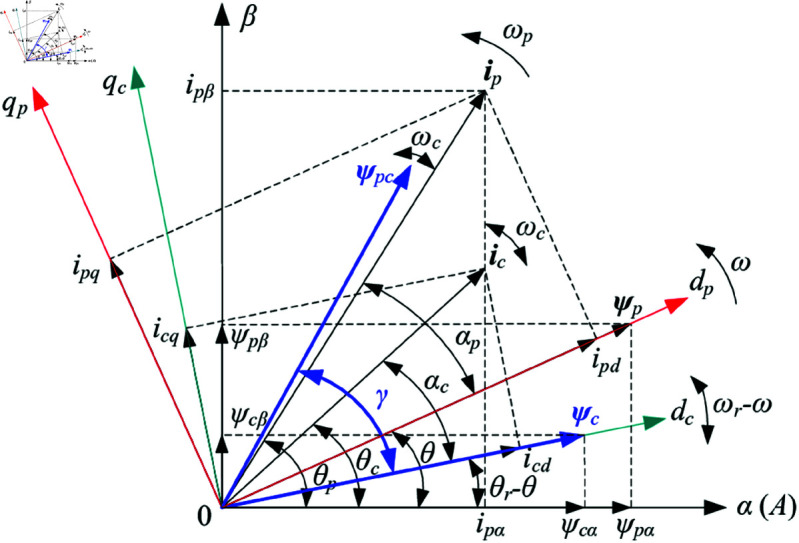
Flux linkage and current vectors corresponding to the reference frames.

In order to better grasp the special operating mechanism of DSBDFWPG (in Figs 1 and 2), and facilitate the system modelling and control, based on magnetic field modulation theory and ideally represented as a unified equivalent circuit of reluctance and cage rotor BDFGs [9,26,35], the space vector mathematical model in three-phase stationary coordinate frame, generating convention for power winding and motoring for control one, respectively, which can be summarized as (8):

$$\left\{ \begin{array}{c} u_{p}=-R_{p}i_{p}+\frac{{d\psi }_{p}}{dt}+j\omega_{p}\psi_{p}\\ u_{c}=R_{c}i_{c}+\frac{{d\psi }_{c}}{dt}+j\omega_{c}\psi_{c} \end{array}\right.$$ (8)

where the relationship among stator inductance, vectors of flux linkage and current in $d_{p,c}\;-\;q_{p,c}$ (or α−β) rotate frames at arbitrary speed *ω* shown in Fig 4 are given as (9)–(10) [7,34]; It should be noted that if $\omega =\omega_{c},\omega_{r}\;-\;\omega_{c}=\omega_{p}$, i.e., the power and control winding reference coordinates are in their own stationary ones, this advantage is very convenient to understand the operating mechanism of DSBDFWPG.

$$\left\{ \begin{array}{c} \psi_{p}=L_{p}i_{p}+L_{pc}i_{pc}\\ \psi_{c}=L_{c}i_{c}+L_{pc}i_{cp} \end{array}\right.$$ (9)

$$\left\{ \begin{array}{c} i_{pc}=i_{c}e^{j\theta_{p}}=i_{c}e^{-j\theta_{c}}e^{j(\theta_{p}+\theta_{c})}=i_{c}^{*}e^{j\theta_{r}}\\ i_{cp}=i_{p}e^{j\theta_{c}}=i_{p}e^{-j\theta_{p}}e^{j(\theta_{p}+\theta_{c})}=i_{p}^{*}e^{j\theta_{r}} \end{array}\right.$$ (10)

where $i_{pc}$ and $i_{cp}$ are the current vectors of the control ($i_{c}$) and power ($i_{p}$) windings “coupling" to the power and control ones, respectively, their frequencies are same as the other windings ($f_{p},f_{c}$), and both of them must via particular magnetic modulation and pole number conversion ($e^{j\theta_{r}}$ is frequency not coordinate transformation) through the specially designed rotor, i.e., ($p_{p},f_{p}$)⇋($p_{c},f_{c}$); $\theta_{p}$, $\theta_{c}$ and $\theta_{r}$ represent the angles of axis between power winding, control, rotor and the same pole number magnetic field of rotor parts, respectively; The superscript “*" denotes the conjugate complex ($i_{c}^{*}$, $i_{p}^{*}$) ; *L*_*pc*_ implies the enhancement of mutual inductance (magnetic modulation) of the specially designed back-to-back cage-barrier rotor.

The peculiar electromagnetic torque production of DSBDFWPG is given as (11):

$$T_{em}=\frac{3p_{r}}{2\sigma L_{c}}|\psi_{pc}\times \psi_{c}|=\frac{3p_{r}}{2\sigma L_{c}}|\psi_{pc}||\psi_{c}|\sin\gamma$$ (11)

where $\sigma =1\;-\;L_{pc}^{2}/(L_{p}L_{c})$ is the leakage factor; *γ* is the torque angle between the mutual flux linkage vector $\psi_{pc}$ and $\psi_{c}$ as shown in Fig 4.

## Fundamentals and optimized DPC for DSBDFWPG

### DPC mechanisms for DSBDFWPG

According to instantaneous reactive power theory, the active and reactive power of power winding are illustrated as (12) [13]:

$$\left\{ \begin{array}{c} cP_{p}=\underset{i_{p\alpha }}{\underbrace{i_{pA}}}\underset{u_{p\alpha }}{\underbrace{\frac{u_{pAB}+u_{pAC}}{2}}}+\underset{i_{p\beta }}{\underbrace{\frac{i_{pA}+2i_{pB}}{\sqrt{3}}}}\underset{u_{p\beta }}{\underbrace{\frac{\sqrt{3}u_{pBC}}{2}}}\\ Q_{p}=\underset{i_{p\alpha }}{\underbrace{i_{pA}}}\underset{u_{p\beta }}{\underbrace{\frac{\sqrt{3}u_{pBC}}{2}}}-\underset{i_{p\beta }}{\underbrace{\frac{i_{pA}+2i_{pB}}{\sqrt{3}}}}\underset{u_{p\alpha }}{\underbrace{\frac{u_{pAB}+u_{pAC}}{2}}} \end{array}\right.$$ (12)

where $u_{p\alpha ,\beta }$ and $i_{p\alpha ,\beta }$ denote the α−β components of power winding voltage and current as shown in Fig 4, respectively; $u_{pAB},u_{pAC},u_{pBC}$ and $i_{pA},i_{pB},i_{pC}$ are line voltage and phase current of power winding, respectively; $i_{pA}+i_{pB}+i_{pC}=0$ for three-phase balanced load.

By combining (2) and (11), the total electromechanical power balance expression can be given as (13):

$$P_{m}=T_{em}\omega_{rm}=\underset{P_{pm}\approx P_{p}}{\underbrace{\frac{T_{em}\omega_{p}}{p_{r}}}}+\underset{P_{cm}\approx P_{c}}{\underbrace{\frac{T_{em}\omega_{c}}{p_{r}}}}=\underset{\approx P_{p}}{\underbrace{P_{pm}}}(1+\frac{\omega_{c}}{\omega_{p}})$$ (13)

where to understand the mechanisms of DPC for DSBDFWPG, one can keep the convention in mind: the larger capacity of generator, the smaller resistance and copper losses of the winding. Thus neglecting the dual-stator copper losses and changes of magnetic energy ($dW/dt\approx d\psi_{p}/dt\approx 0$) due to power winding directly grid-connected, the mechanical power components of dual-stator windings are probably equal to their active power counterparts, i.e., $P_{m}=P_{pm}\;+\;P_{cm}$, $P_{pm}\approx P_{p}$, $P_{cm}\approx P_{c}$. Noting that for generating convention *T*_*em*_<0 and *P*_*pm*_ (also *P*_*p*_) < 0, the power winding always supply active power to the grid; But for the control winding, *P*_*cm*_ (*P*_*c*_) > 0 for absorbing from the grid in sub-synchronous and *P*_*cm*_ (*P*_*c*_) < 0 supplying active power to the grid in super-synchronous operation mode. As a result, a back-to-back four-quadrant converter should be adopted for bidirectional power flow in the control winding, i.e., MSC and GSC as shown in Fig 1. Therefore, one can implement the active power control of DPC according to the relationship: $\gamma \to T_{em}\to P_{m}\to P_{pm}\to P_{p}$.

For the reactive power control of DPC, which can be explained by the total magnetic field/flux balance of conventional doubly-fed generator(s): Assuming that the total magnetic field is kept constant, if one winding “contributes" more (or less) magnetic field/flux, the other winding can “contribute" less (or more) field/flux. Therefore, considering that the excitation magnetic field/flux is directly proportional to the exciting current and reactive power, the reactive power of the power winding can be indirectly decreased (or increased) by increasing (or decreasing) the reactive power of the control winding (i.e., contributing more/less magnetic field/flux), and even the UPFC can be realized by setting reference value of reactive power to zero (i.e., $Q_{p}^{ref}=0$) with control winding “contributing" all the dual-stator magnetic field/flux of DSBDFWPG.

### Effects of voltage space vectors on power variations

**Fig 5:**
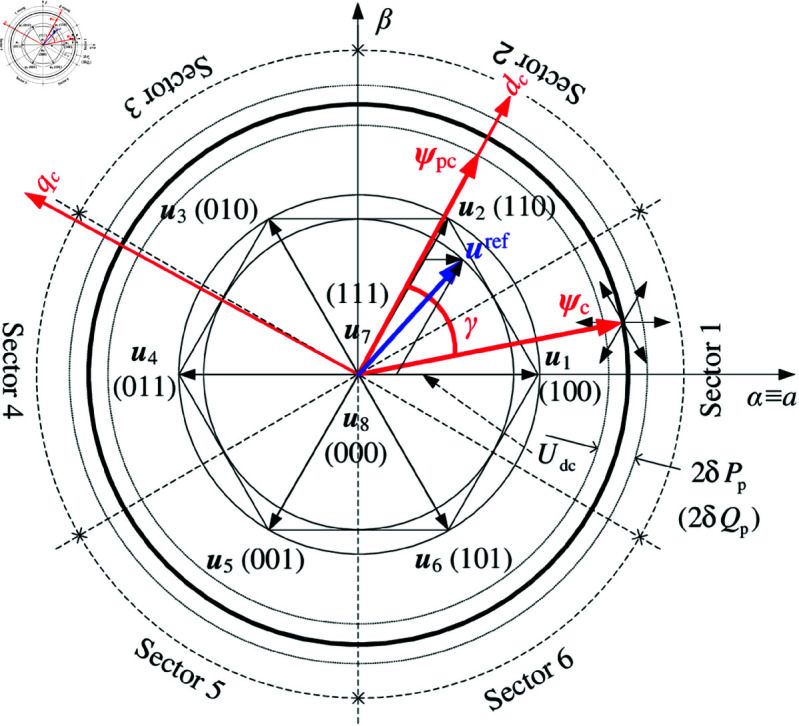
Flux linkage, voltage space vectors and sector division of MSC.

The vectors of flux linkage $\psi_{pc}$ and $\psi_{c}$, voltage space vectors ($u_{1}\;-\;u_{8},u^{ref}$) and sectors 1–6 division of MSC (in Fig 1) are shown in Fig 5.

where the effective vectors $u_{1}\;-\;u_{6}$ are located at the vertex of regular hexagon, zero vectors $u_{7}$ and $u_{8}$ at the origin of coordinate, respectively; DC bus and reference voltage vector of SVPWM are *U*_*dc*_ and $u^{ref}$; The boundary of sectors 1–6 are the angular bisector of regular hexagon; The half width of power hysteresis comparators are $\delta P_{p}$ and $\delta Q_{p}$, respectively. Assuming that $\psi_{pc}$ rotates counterclockwise at $\omega_{c}$ and aligns with *d*_*c*_ axis, which is ahead of the $\psi_{c}$ with torque angle *γ* for generating convention, $\psi_{c}$ is located in sector 1, then the *γ* will be decreased/increased with $u^{ref}$ leading/lagging $\psi_{c}$ by $0\;-\;180^{\circ }$, and the active power (*P*_*p*_) can be controlled via $u^{ref}\to \gamma \to T_{em}\to P_{p}$. When the absolute angle between $u^{ref}$ and $\psi_{c}$ is less/greater than $90^{\circ }$, the amplitude of $\psi_{c}$ ($\left|\psi_{c}\right|$) and reactive power (*Q*_*c*_) contributed by the control winding will be increased/decreased, then the reactive power (*Q*_*p*_) and magnetic field/flux contributed by power winding will be indirectly decreased/increased via $u^{ref}\to |\psi_{c}|\to Q_{c}\to |\psi_{p}|\to Q_{p}$.

In conclusion, one can summarize the intrinsic mechanisms of DPC for DSBDFWPG as: if both the active and reactive power of the power winding need to be controlled simultaneously, the $u_{1}\;-\;u_{6}$ for LUT-DPC (or $u^{ref}$ for SVPWM) with the appropriate frequency and rotating direction (i.e., the control winding exciting/field current phase sequence) in a sector should be correctly selected in time. The relationship among effective vectors of $u_{1}\;-\;u_{6}$ in MSC, trend of reactive power (increased/decreased) and sectors 1–6 changes ($\psi_{c}$ rotates anticlockwise from sector 1 to 6 or clockwise from 6 to 1) for LUT-DPC can be summed up as Table 1 [22,23].

**Table 1:** Relationship among the vector, trend of reactive power and sector changes.

Sector	$u_{1}$	$u_{2}$	$u_{3}$	$u_{4}$	$u_{5}$	$u_{6}$
1	*x*	−|−1	+|+1	*x*	+|−1	−|+1
2	−|+1	*x*	−|−1	+|+1	*x*	+|−1
3	+|−1	−|+1	*x*	−|−1	+|+1	*x*
4	*x*	+|−1	−|+1	*x*	−|−1	+|+1
5	+|+1	*x*	+|−1	−|+1	*x*	−|−1
6	−|−1	+|+1	*x*	+|−1	−|+1	*x*

Where “*x*" indicates that a vector of $u_{1}-u_{6}$ is discarded due to it will cause reactive power to change too drastically; “+/-" represents that the vector will cause the change rate of reactive power to be increased/decreased, respectively; “+1/-1" shows that the sector will be circularly increased from 1 to 6 or decreased from 6 to 1.

### Optimized DPC based on back-stepping method

Back-stepping method can be used to design the systematic feedback controller for non-linear and/or uncertain parameters systems [36–40], its main idea is to decompose the complex non-linear system into multiple low order subsystems which do not exceed the highest order of the original one, and design the related Lyapunov function for each subsystem from lower to higher order step by step. According to the Lyapunov theorem of asymptotic stability and intermediate virtual control quantities, the control law of whole feedback system can be finally deduced.

The dual-stator winding current vectors in *α*-*β* coordinate can be given as (14), the apparent power and its derivative are shown as (15), (16):

$$\left\{ \begin{array}{c} \frac{di_{p\alpha \beta }}{dt}=\frac{1}{\sigma L_{p}}[\left(u_{p\alpha \beta }+R_{p}i_{p\alpha \beta }\right)-j\left(\omega_{p}L_{p}+\omega_{c}\frac{L_{pc}^{2}}{L_{c}}\right)i_{p\alpha \beta }\;-\frac{L_{pc}}{L_{c}}{\left(u_{c\alpha \beta }-R_{c}i_{c\alpha \beta }\right)}^{*}-j\omega_{r}L_{pc}i_{c\alpha \beta }^{*}]\;\frac{di_{c\alpha \beta }}{dt}=-\frac{L_{pc}}{\sigma L_{p}L_{c}}[{\left(u_{p\alpha \beta }+R_{p}i_{p\alpha \beta }\right)}^{*}+j\left(\omega_{p}L_{pc}+\omega_{c}\frac{L_{p}L_{c}}{L_{pc}}\right)i_{c\alpha \beta }^{*}\;-\frac{L_{p}}{L_{pc}}{\left(u_{c\alpha \beta }-R_{c}i_{c\alpha \beta }\right)}^{*}-j\omega_{r}L_{p}i_{p\alpha \beta }^{*}]\; \end{array}\right.$$ (14)

$$S_{p}=P_{p}+jQ_{p}=\frac{3}{2}u_{p\alpha \beta }i_{p\alpha \beta }^{*}$$ (15)

$$\frac{dS_{p}}{dt}=\frac{3}{2}\left(\frac{du_{p\alpha \beta }}{dt}i_{p\alpha \beta }^{*}+u_{p\alpha \beta }\frac{di_{p\alpha \beta }^{*}}{dt}\right)$$ (16)

In view of the power winding directly grid-connected, the derivative term of its voltage can be given as (17):

$$\frac{du_{p\alpha \beta }}{dt}=\frac{d}{dt}\left(\|u_{p\alpha \beta }\|e^{j\omega_{p}}\right)=j\omega_{p}\|u_{p\alpha \beta }\|e^{j\omega_{p}}=j\omega_{p}u_{p\alpha \beta }$$ (17)

Substitute (14), (17) into (16) and decompose it into real and imaginary parts as (18):

$$\left\{ \begin{array}{c} \frac{dP_{p}}{dt}=\frac{R_{p}}{\sigma L_{p}}P_{p}-\left(\omega_{p}\frac{1+\sigma }{\sigma }+\omega_{c}\frac{1-\sigma }{\sigma }\right)Q_{p}\;+\frac{3L_{pc}}{2\sigma L_{p}}\left[\left(\frac{R_{c}}{L_{c}}u_{p\alpha }-\omega_{r}u_{p\beta }\right)i_{c\alpha }-\left(\frac{R_{c}}{L_{c}}u_{p\beta }+\omega_{r}u_{p\alpha }\right)i_{c\beta }\right]\;-\frac{3L_{pc}}{2\sigma L_{p}L_{c}}\left(u_{p\alpha }u_{c\alpha }-u_{p\beta }u_{c\beta }\right)+\frac{3}{2\sigma L_{p}}\|u_{p\alpha \beta }{\|}^{2}\;\frac{dQ_{p}}{dt}=\frac{R_{p}}{\sigma L_{p}}Q_{p}+\left(\omega_{p}\frac{1+\sigma }{\sigma }+\omega_{c}\frac{1-\sigma }{\sigma }\right)P_{p}\;+\frac{3L_{pc}}{2\sigma L_{p}}\left[\left(\frac{R_{c}}{L_{c}}u_{p\beta }+\omega_{r}u_{p\alpha }\right)i_{c\alpha }+\left(\frac{R_{c}}{L_{c}}u_{p\alpha }-\omega_{r}u_{p\beta }\right)i_{c\beta }\right]\;-\frac{3L_{pc}}{2\sigma L_{p}L_{c}}\left(u_{p\beta }u_{c\alpha }+u_{p\alpha }u_{c\beta }\right)\; \end{array}\right.$$ (18)

where the power derivative terms are only first-order, which can be directly used to design the proposed back-stepping-based direct power controller by Lyapunov theorem of asymptotic stability.

The active and reactive power deviations are defined as (19):

$$\left\{ \begin{array}{c} e_{P}=P_{p}^{ref}-P_{P}\\ e_{Q}=Q_{p}^{ref}-Q_{P} \end{array}\right.$$ (19)

where $P_{p}^{ref}$ and $Q_{p}^{ref}$ denote the power winding reference active and reactive power values, respectively. Obviously, if the deviations of *e*_*P*_ and *e*_*Q*_ are asymptotically stable at 0, the *P*_*p*_ and *Q*_*p*_ will converge to $P_{p}^{ref}$ and $Q_{p}^{ref}$, respectively, then the desired active and reactive power tracking control can be implemented.

The positively definite Lyapunov function V is given as (20), substitute (18) and (19) into its derivative and shown as (21):

$$V=\frac{1}{2}e_{P}^{2}+\frac{1}{2}e_{Q}^{2}\geq 0$$ (20)

$$\frac{dV}{dt}=-e_{P}\frac{dP_{p}}{dt}-e_{Q}\frac{dQ_{p}}{dt}\;=-e_{P}\{\frac{R_{p}}{\sigma L_{p}}P_{p}-\left(\omega_{p}\frac{1+\sigma }{\sigma }+\omega_{c}\frac{1-\sigma }{\sigma }\right)Q_{p}\;+\frac{3L_{pc}}{2\sigma L_{p}}\left[\left(\frac{R_{c}}{L_{c}}u_{p\alpha }-\omega_{r}u_{p\beta }\right)i_{c\alpha }-\left(\frac{R_{c}}{L_{c}}u_{p\beta }+\omega_{r}u_{p\alpha }\right)i_{c\beta }\right]\;-\frac{3L_{pc}}{2\sigma L_{p}L_{c}}\left(u_{p\alpha }u_{c\alpha }-u_{p\beta }u_{c\beta }\right)+\frac{3}{2\sigma L_{p}}\|u_{p\alpha \beta }{\|}^{2}\}\;-e_{Q}\{\frac{R_{p}}{\sigma L_{p}}Q_{p}+\left(\omega_{p}\frac{1+\sigma }{\sigma }+\omega_{c}\frac{1-\sigma }{\sigma }\right)P_{p}\;+\frac{3L_{pc}}{2\sigma L_{p}}\left[\left(\frac{R_{c}}{L_{c}}u_{p\beta }+\omega_{r}u_{p\alpha }\right)i_{c\alpha }+\left(\frac{R_{c}}{L_{c}}u_{p\alpha }-\omega_{r}u_{p\beta }\right)i_{c\beta }\right]\;-\frac{3L_{pc}}{2\sigma L_{p}L_{c}}\left(u_{p\beta }u_{c\alpha }+u_{p\alpha }u_{c\beta }\right)\}\;$$ (21)

In order to make (20) satisfy the Lyapunov theorem of asymptotic stability, (21) can be reconstructed as (22):

$$\frac{dV}{dt}=-k_{P}e_{P}^{2}-k_{Q}e_{Q}^{2}$$ (22)

where *k*_*P*_ and *k*_*Q*_ are the positive constants to be determined.

Taking the integral of (22) and considering the positive (20), which can be shown as (23):

$$\left\{ \begin{array}{c} \int_{0}^{\infty }\frac{dV}{dt}\;dt=V\left(\infty \right)-V\left(0\right)\leq 0\\ V\left(\infty \right)\geq 0 \end{array}\right.\;\Rightarrow -V\left(0\right)\leq V\left(\infty \right)-V\left(0\right)\leq 0$$ (23)

According to Barbalat’s lemma [36], (23) exists and is bounded, then its limit value can be obtained as (24):

$$\lim_{t\to \infty }\frac{dV}{dt}=0\Rightarrow \left\{ \begin{array}{c} \lim_{t\to \infty }e_{P}=0\\ \lim_{t\to \infty }e_{Q}=0 \end{array}\right.$$ (24)

The back-stepping-based direct power controller designed above can make the system power deviation *e*_*P*_ and *e*_*Q*_ asymptotically stable at 0, that is, the *P*_*p*_ and *Q*_*p*_ will converge to the $P_{p}^{ref}$ and $Q_{p}^{ref}$ in steady state, respectively.

According to (21), (22), the BS-DPC control law under Lyapunov theorem of asymptotic stability can be obtained as (25):


$$\left\{ \begin{array}{c} k_{P}e_{P}=\frac{dP_{p}}{dt}\\ k_{Q}e_{Q}=\frac{dQ_{p}}{dt} \end{array}\right.\Rightarrow \;$$

$$\left\{ \begin{array}{c} u_{c\alpha }=\frac{2\sigma L_{p}L_{c}}{3\|u_{p\alpha \beta }{\|}^{2}L_{pc}}[-k_{P}\left(P_{p}^{ref}-P_{p}\right)u_{p\alpha }-k_{Q}\left(Q_{p}^{ref}-Q_{p}\right)u_{p\beta }\;+\frac{R_{p}}{\sigma L_{p}}\left(P_{p}u_{p\alpha }+Q_{p}u_{p\beta }\right)\;-\left(\omega_{p}\frac{1+\sigma }{\sigma }+\omega_{c}\frac{1-\sigma }{\sigma }\right)\left(Q_{p}u_{p\alpha }-P_{p}u_{p\beta }\right)]\;+\left(R_{c}i_{c\alpha }-\omega_{r}L_{c}i_{c\beta }\right)+\frac{L_{c}}{L_{pc}}u_{p\alpha }\;u_{c\beta }=\frac{2\sigma L_{p}L_{c}}{3\|u_{p\alpha \beta }{\|}^{2}L_{pc}}[k_{P}\left(P_{p}^{ref}-P_{p}\right)u_{p\beta }-k_{Q}\left(Q_{p}^{ref}-Q_{p}\right)u_{p\alpha }\;+\frac{R_{p}}{\sigma L_{p}}\left(Q_{p}u_{p\alpha }-P_{p}u_{p\beta }\right)\;+\left(\omega_{p}\frac{1+\sigma }{\sigma }+\omega_{c}\frac{1-\sigma }{\sigma }\right)\left(P_{p}u_{p\alpha }+Q_{p}u_{p\beta }\right)]\;+\left(R_{c}i_{c\beta }+\omega_{r}L_{c}i_{c\alpha }\right)-\frac{L_{c}}{L_{pc}}u_{p\beta }\; \end{array}\right.$$ (25)

**Fig 6:**
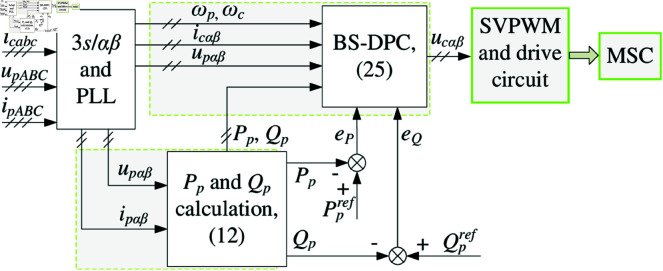
Diagram of the proposed BS-DPC.

The proposed BS-DPC for DSBDFWPG can be shown as Fig 6. Where including 3s/αβ coordinate transformation of voltage and current from three-phase stationary to α−β coordinate; Phase lock loop (PLL) calculation of $\omega_{p}$, then $\omega_{c}$ can be obtained; *P*_*p*_ and *Q*_*p*_ calculation as (12); BS-DPC controller as (25); Based on SVPWM, the IGBTs of MSC are driven with a fixed switching frequency, which can reduce power fluctuations and current THD of power winding.

## Simulation and experimental results

**Fig 7:**
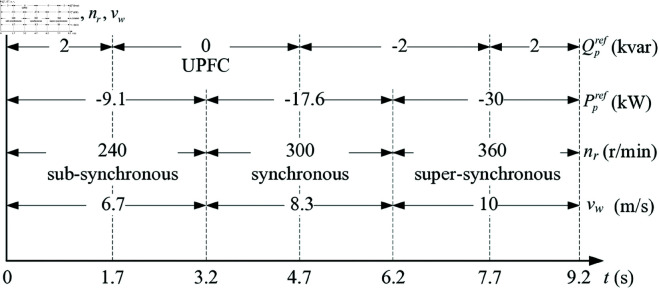
The reference values.

The ratings and parameters of 12/8-pole 50 kW DSBDFWPG prototype tested off-line are given in Table 2, the control system reference values of LUT-DPC and optimized BS-DPC for VSCF-MPPT WPGS are shown as Fig 7.

**Table 2:** The DSBDFWPG ratings and parameters.

Parameters	Values
Rated power (kW)	*P*_*N*_ = 50
Rated stator voltage (V)	*U*_*p*_ = 380, *U*_*c*_ = 380
Rated stator frequency (Hz)	*f*_*p*_ = 50, *f*_*c*_ = 50
Stator winding resistance (Ω)	*R*_*p*_ = 0.1653, *R*_*c*_ = 0.2617
Stator winding inductance (mH)	*L*_*p*_ = 16.20, *L*_*c*_ = 49.70
Mutual inductance (mH)	*L*_*pc*_ = 22.79
Stator and rotor poles	*p*_*p*_ = 6, *p*_*c*_ = 4, *p*_*r*_ = 10
Synchronous speed (r/min)	*n*_*syn*_ = 300

### Simulation results of LUT-DPC and BS-DPC

To make the simulation similar to the real conditions, the Gaussian distributed random signal is superimposed on the given ideal velocity to simulate the deviation and integration errors of voltage/current, speed sensors and other actual measurements noise. Setting the sampling frequency to 20 kHz, the active and reactive power hysteresis width as $\delta P_{p}=\pm 0.3$ kW, $\delta Q_{p}=\pm 0.3$ kvar, respectively.

**Fig 8:**
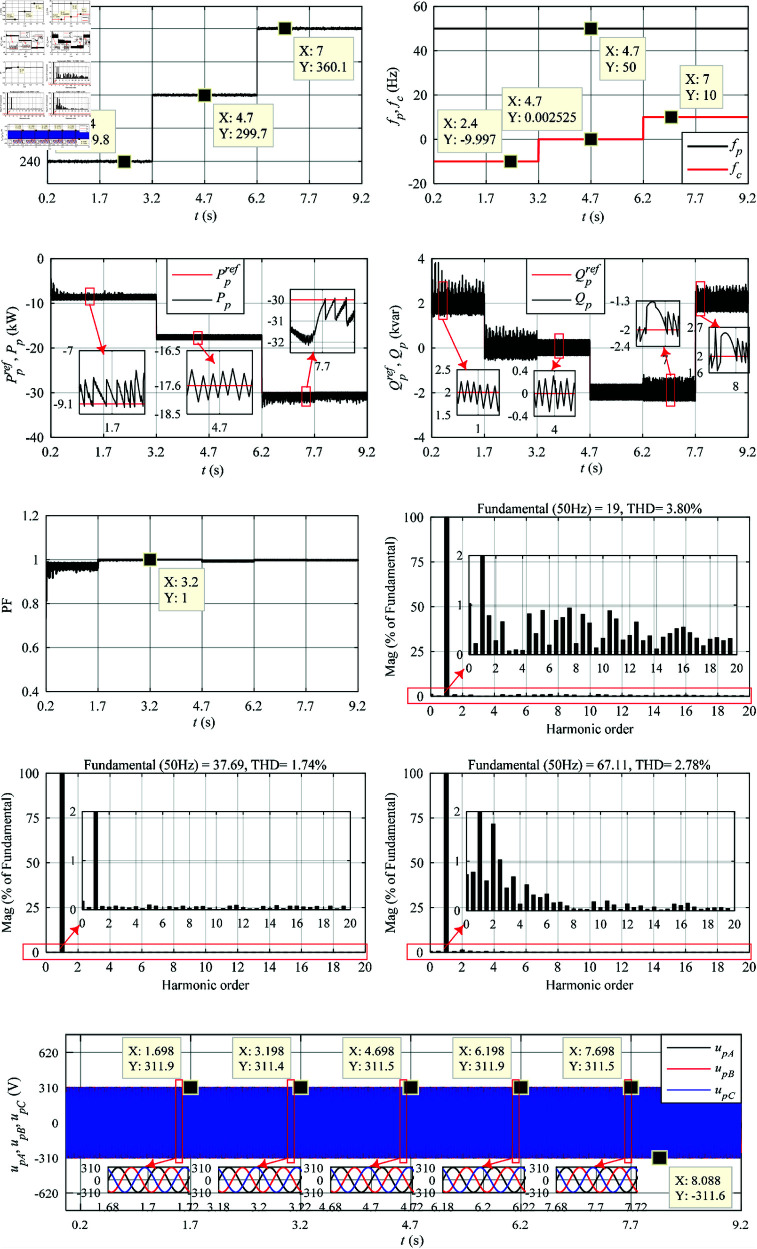
Simulation results of the traditional LUT-DPC. (a) *n*_*r*_. (b) *f*_*p*_ and *f*_*c*_. (c) $P_{p}^{ref}$ and *P*_*p*_. (d) $Q_{p}^{ref}$ and *Q*_*p*_. (e) Power factor in power winding. (f) FFT of *i*_*pA*_ in 1–1.04 s, THD = 3.80%. (g) FFT of *i*_*pA*_ in 4–4.04 s, THD = 1.74%. (h) FFT of *i*_*pA*_ in 7–7.04 s, THD = 2.78%. (i) Three-phase voltage (amplitude) of the power winding.

**Fig 9:**
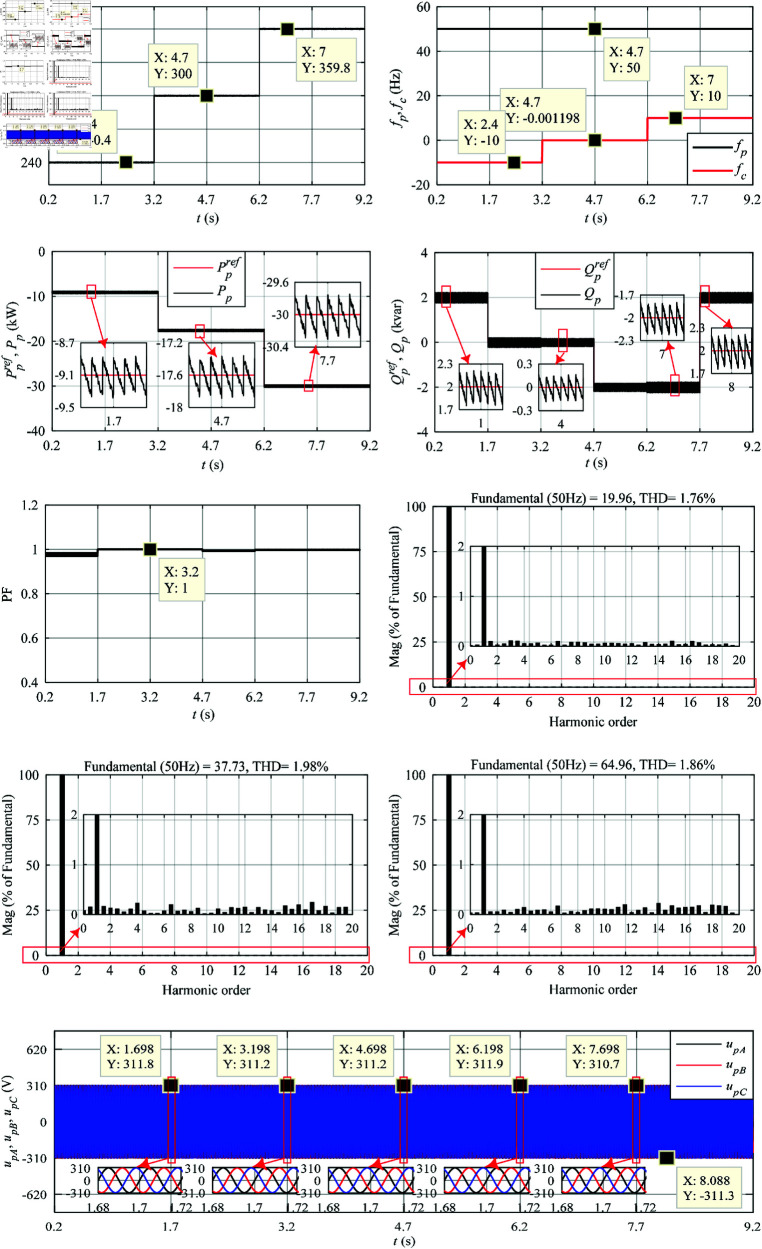
Simulation results of the proposed BS-DPC. (a) *n*_*r*_. (b) *f*_*p*_ and *f*_*c*_. (c) $P_{p}^{ref}$ and *P*_*p*_. (d) $Q_{p}^{ref}$ and *Q*_*p*_. (e) Power factor in power winding. (f) FFT of *i*_*pA*_ in 1–1.04 s, THD = 1.76%. (g) FFT of *i*_*pA*_ in 4–4.04 s, THD = 1.98%. (h) FFT of *i*_*pA*_ in 7–7.04 s, THD = 1.86%. (i) Three-phase voltage (amplitude) of the power winding.

Based on MATLAB/Simulink^®^ and SimPowerSystems^®^ toolboxes, the performance analysis of LUT-DPC (in Table 1 [23]) and optimized BS-DPC (in Fig 6) strategies for DSBDFWPG are illustrated in Figs 8 and 9, which including the characteristics of VSCF, active power control with MPPT, reactive power control and UPFC, THD of *i*_*pA*_ with fast fourier transform (FFT) at different speed and power reference values shown in Fig 7. Besides, the voltage waveforms of the power winding are also presented to verify the variable-speed constant-frequency and constant-voltage power generation characteristics of this generator system.

To save space, the comparative analysis of simulation and validated experimental results of the LUT-DPC and BS-DPC are comprehensively discussed in the subsection of experimental tests.

### Experimental tests of LUT-DPC and BS-DPC

**Fig 10:**
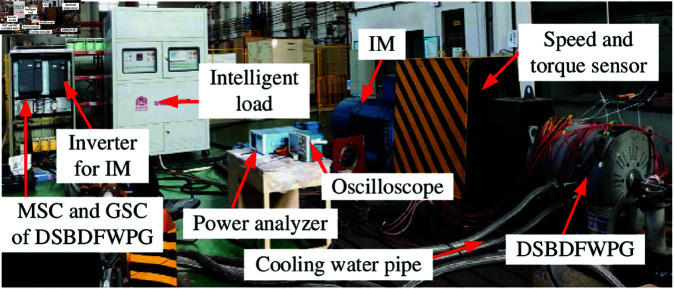
Experimental test rigs.

The experimental test rigs of DSBDFWPG for LUT-DPC and BS-DPC in VSCF-MPPT WPGS are shown in Fig 10, where the prime mover is adopted a normal inverter-fed induction motor (IM), which simulates the operation of a wind turbine. The specially customized control winding back-to-back four-quadrant two-level converter (MSC and GSC) is implemented, its rated capacity is 30 kW, IGBT is selected as FF75R12RT4. The DC bus adopts a copper bar design to optimize the electrical performance. The DC capacitor is composed of two B43310-S9398-M1 electrolytic capacitors (3900 *μ*F/400 V) connected in series, and the matching voltage equalizing resistor selected is HPP150-4.7KJX3. The pre-charging stage is equipped with A 30 Ω/80 W current-limiting resistor, and the charging control adopts Schneider LC1D38BD contactor (rated value 690 V/50 A). The IGBT module is connected in parallel with the B32656-Y7105-K210 capacitor (1*μ*F/1200V) to absorb the peak voltage of the switch. The temperature detection adopts the NTC-103F3950 thermistor, and the current detection uses the LT58-S7 sensor of LEM Company (transformation ratio 1000:1). The controller algorithm is implemented based on two TI TMS320LF28335 for MSC and GSC, respectively.

**Fig 11:**
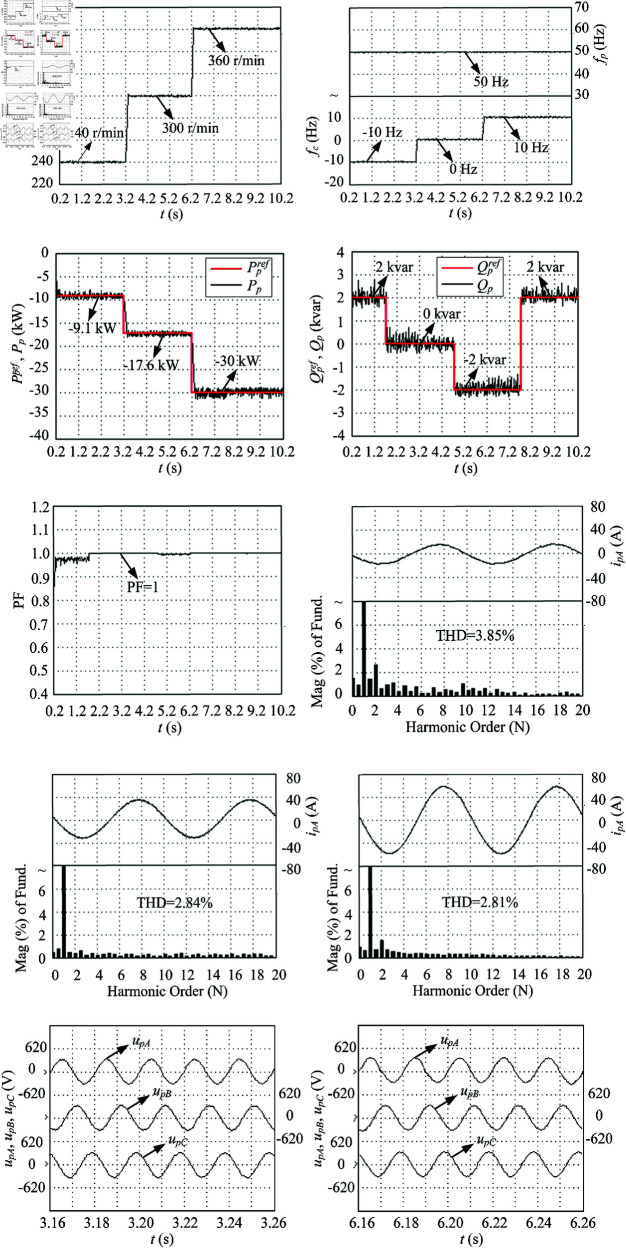
Experimental tests of the traditional LUT-DPC. (a) *n*_*r*_. (b) *f*_*p*_ and *f*_*c*_. (c) $P_{p}^{ref}$ and *P*_*p*_. (d) $Q_{p}^{ref}$ and *Q*_*p*_. (e) Power factor in power winding. (f) FFT of *i*_*pA*_ in 1–1.04 s, THD = 3.85%. (g) FFT of *i*_*pA*_ in 4–4.04 s, THD = 2.84%. (h) FFT of *i*_*pA*_ in 7–7.04 s, THD = 2.81%. (i) Three-phase voltage (amplitude) of the power winding in sub-synchronous operation. (j) Three-phase voltage (amplitude) of the power winding in super-synchronous operation.

**Fig 12:**
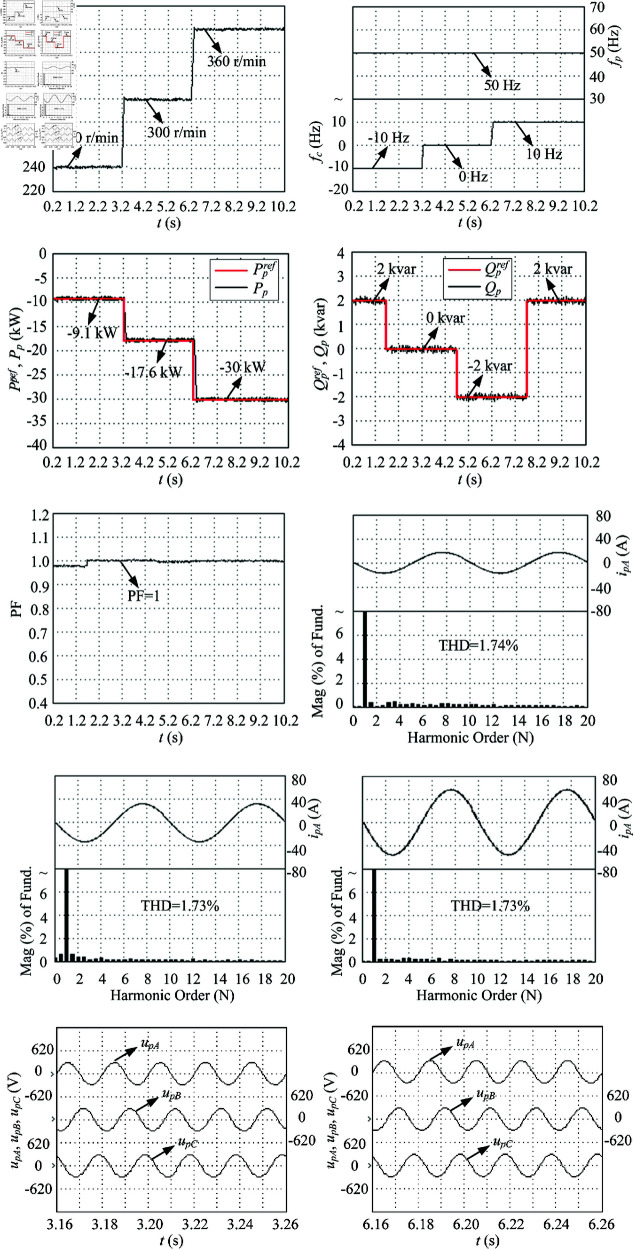
Experimental tests of the proposedc BS-DPC. (a) *n*_*r*_. (b) *f*_*p*_ and *f*_*c*_. (c) $P_{p}^{ref}$ and *P*_*p*_. (d) $Q_{p}^{ref}$ and *Q*_*p*_. (e) Power factor in power winding. (f) FFT of *i*_*pA*_ in 1–1.04 s, THD = 3.85%. (g) FFT of *i*_*pA*_ in 4–4.04 s, THD = 2.84%. (h) FFT of *i*_*pA*_ in 7–7.04 s, THD = 2.81%. (i) Three-phase voltage (amplitude) of the power winding in sub-synchronous operation. (j) Three-phase voltage (amplitude) of the power winding in super-synchronous operation.

The performance experimental tests of LUT-DPC (as Table 1 [23]) and BS-DPC (as Fig 6) strategies for DSBDFWPG are illustrated in Figs 11 and 12, which follow the conditions shown in Fig 7 and corresponding to the above simulation results in Figs 8 and 9, respectively.

As shown in Figs 8, 9, 11, 12(a), and 12(b), when the step change wind velocity ($v_{w}$) is 6.7 m/s, 8.3 m/s and 10 m/s (rated value) with *C*_*p*_ = 0.48, λ=8.1 and β=0 for maximum power capturing of wind turbine (as shown in Fig 3), the step change *n*_*r*_ is about 240 r/min (sub-synchronous), 300 r/min (synchronous) and 360 r/min (super-synchronous), which corresponding to the rated parameter variation range of DSBDFWPG (i.e., *f*_*c*_ within ±10 Hz). The power and control winding frequencies ($f_{p},f_{c}$) are kept as 50 Hz of the grid, −10 Hz (sub-synchronous), 0 Hz (synchronous) and 10 Hz (super-synchronous) referring to (1), respectively. Where the negative/positive frequency (*f*_*c*_ = −10 Hz,10 Hz) denotes that the exciting/field current phase sequence of control winding is opposite/same to that of power one for sub/super-synchronous mode, respectively, and *f*_*c*_ = 0 Hz for DC exciting in synchronous operation. Obviously, the VSCF characteristic for DSBDFWPG is well fulfilled in LUT-DPC and BS-DPC.

As shown in Figs 8, 9, 11, 12(c), 12(d), and 12(e), the active power (*P*_*p*_) can track the $P_{p}^{ref}$ (MPPT) with –9.1 kW, –17.6 kW and –30 kW, meanwhile the reactive power (*Q*_*p*_) can track the given step change values with $Q_{p}^{ref}$ as 2 kvar, 0 kvar, –2 kvar and 2 kvar, respectively. Where the negative active/reactive powers only denote that the power winding is fed power to the grid. Apparently, the power fluctuations of proposed BS-DPC are kept within the setting threshold values of ±0.3 kW and ±0.3 kvar, which overwhelming to the traditional LUT-DPC (exceeds the setting threshold values, about –0.3 kW–+1 kW and ±0.5 kvar, respectively). The main reason is that the inherent drawbacks of bang-bang control in traditional LUT-DPC, i.e., only the effective voltage space vectors ($u_{1}\;-\;u_{6}$) are adopted without zero ones ($u_{7},u_{8}$). However the proposed BS-DPC is designed based on Lyapunov theorem of asymptotic stability and SVPMW technology, the $u^{ref}$ is combined with the effective ($u_{1}\;-\;u_{6}$) and zero vectors ($u_{7},u_{8}$) adopted in seven segment SVPWM, and the $u^{ref}$ can be at any location of sectors 1–6 not just the six fixed locations ($u_{1}\;-\;u_{6}$), which completely superior to LUT-DPC. In 1.7 – 4.7 s, the *Q*_*p*_ tracks $Q_{p}^{ref}=0$, power factor ≈ 1, then UPFC of LUT-DPC and BS-DPC is effectively implemented.

During the steady state with different speed (operation mode) and active/reactive power: 1 - 1.04 s with *n*_*r*_ = 240 r/min (sub-synchronous), *P*_*p*_ = -9.1 kW and *Q*_*p*_ = 2 kvar; 4 - 4.04 s with *n*_*r*_ = 300 r/min (synchronous), *P*_*p*_ = -17.6 kW and *Q*_*p*_ = 0 kvar; 7 - 7.04 s with *n*_*r*_ = 360 r/min (super-synchronous), *P*_*p*_ = -30 kW and *Q*_*p*_ =  -2 kvar; The THD of simulation and experimental results for LUT-DPC are 3.80%, 1.74%, 2.78% and 3.85%, 2.84%, 2.81%, respectively; However, for the proposed BS-DPC, THD is less than that of LUT-DPC in general, which only about 1.76%, 1.98%, 1.86% and 1.74%, 1.73%, 1.73%, respectively.

As shown in Figs 8(i), 9(i), 11(i), 11(j), 12(i), and 12(j), the voltage amplitude of DSBDFWPG is 311 V (i.e., the effective value is 220 V, the line value is 380 V). It can be seen that both LUT-DPC and BS-DPC can achieve good characteristics of variable speed constant frequency and constant voltage power generation control.

By comparing the comprehensive simulation and experimental results above, one can observe some outstanding performances of the proposed BS-DPC over traditional LUT-DPC, the primary advantages including but not limited to the preferable MPPT of active, reactive power control with minor power fluctuations of *P*_*p*_ and *Q*_*p*_, UPFC, the smaller THD and power winding voltages, as Table 3.

**Table 3:** Comparison of simulation and experimental results characteristics of LUT-DPC and BS-DPC.

Metric	LUT-DPC	BS-DPC
Rotor speed (r/min)	240, 300, 360	240, 300, 360
Power winding frequency (Hz)	50	50
Power winding frequency (Hz)	–10, 0, 10	–10, 0, 10
Active power (kW)	–9.1, –17.6, –30	–9.1, –17.6, –30
Errors of active power (kW)	–0.3–+1	± 0.3
Reactive power (kvar)	2, 0, –2, 2	2, 0, –2, 2
Errors of reactive power (kvar)	±0.5	± 0.3
UPFC	1	1
THD (%)	3.80%, 1.74%, 2.78% for simulation 3.85%, 2.84%, 2.81% for exam	1.76%, 1.98%, 1.86% for simulation 1.74%, 1.73%, 1.73 for exam
Power winding voltage (V)	311 in amplitude (220 for RMS, 380 for line value)	311 in amplitude (220 for RMS, 380 for line value)

## Conclusions

In this paper, a novel back-stepping-based direct power control (BS-DPC) for an emerging dual-stator brushless doubly-fed wind power generator (DSBDFWPG) has been presented. The DSBDFWPG consists of inner/outer power/control windings, which are coupled with a specially designed back-to-back cage-barrier rotor. The two stators are connected in series to ensure electromagnetic consistency of inner/outer same-phase power/control winding, respectively. The mathematical model in the form of space vectors and mechanisms of VSCF maximum power point tracking (MPPT) are proposed. By analyzing the comprehensive simulation and experimental results, the correctness, feasibility and effectiveness of the proposed BS-DPC overwhelming to traditional look-up table direct power control (LUT-DPC) have been validated, where including the characteristics of VSCF, MPPT, reactive power and unit power factor control, total harmonic distortion (THD) and power winding voltage. This paper provides a more robust foundation and reference for subsequent control strategies (with or without speed/position sensors), particularly in the context of parameter perturbations and other robustness considerations.
